# Creating Clinical Spaces of Presence: A Phenomenological Study of Nurse Managers’ Ward Rounds in Japan

**DOI:** 10.1155/jonm/8569470

**Published:** 2026-10-15

**Authors:** Yoshie Tejima, Maki Taniyama

**Affiliations:** ^1^ Department of Nursing School of Health Sciences at Odawara International University of Health and Welfare Odawara Japan iuhw.ac.jp

**Keywords:** embodied perception, nurse managers, nursing management, phenomenology, ward rounds

## Abstract

**Aim(s):**

To explore and describe nurse managers’ everyday practices during ward rounds in Japanese hospital settings, focusing on how perception guides practice within unfolding clinical situations.

**Background:**

Although nurse managers increasingly assume administrative responsibilities, they continue to maintain a clinical presence through ward rounds. Little is known about how nurse managers engage with unfolding situations and how such engagement shapes practice.

**Method(s):**

A phenomenologically informed qualitative design drawing on Merleau–Ponty’s theory of perception was employed. Repeated participant observations and interviews were conducted with five nurse managers. Data from all five participants were included in the initial analysis, with the final in‐depth phenomenological analysis focusing on three participants for whom the relationship between perception and practice could be analytically traced across the observational and interview data.

**Results:**

Four themes were identified: Creating Clinical Spaces of Presence, Responding Through the Body, Reflecting in the Moment, and Demonstrating Managerial Presence. Across these themes, the ward rounds examined were understood as situated and relational practices in which perception, embodied responsiveness, reflection, and managerial responsibility were intertwined.

**Conclusion(s):**

The findings suggest that, in the cases examined, ward rounds may involve more than routine surveillance, encompassing ongoing relational responsiveness and the enactment of managerial responsibility within everyday care. These practices suggest that nurse managers may sustain the relational world of the ward through their engagement with patients and clinical situations.

**Implications for Nursing Management:**

Professional development for nurse managers may benefit from supporting perceptual sensitivity, embodied responsiveness, and reflective capacity during ward rounds, in addition to decision‐making and coordination.

## 1. Introduction

Nurse managers play a critical role in shaping the quality of nursing practice and patient care within hospital wards [1]. Although they are often positioned as administrative leaders responsible for staffing, coordination, and organizational outcomes, they also remain present in clinical settings through everyday practices, such as ward rounds. During rounds, nurse managers encounter patients directly, attend to the ward atmosphere, and engage with emerging clinical concerns, creating opportunities through which relationships and clinical meanings may become visible within the ward. Nurse managers’ visibility in clinical settings represents an important expression of leadership presence, through which they may influence staff engagement, caring, and patient outcomes [2].

In Japan, as in many countries, the nurse manager role has increasingly shifted toward managerial and organizational responsibilities. Despite these competing demands, maintaining direct engagement with patients remains an important part of nurse manager practice, often through ward rounds in which they enter patient rooms and interact with patients and families. Such direct clinical engagement reflects the relational and attentive nature of nursing practice [3]. However, little is known about how nurse managers experience and respond to unfolding situations during ward rounds.

Nurse managers’ rounding practices emerge through ongoing engagement with patients, staff, and the ward environment, requiring responsiveness to changing situations and concerns. These practices cannot be fully understood through retrospective accounts of decision‐making or standardized descriptions of managerial behaviors alone. Although reflective accounts provide valuable insights into professional learning [4], they do not fully capture how practice unfolds in the immediacy of clinical encounters. Understanding such practices therefore requires attention to how nurse managers perceive and respond to situations as they arise, particularly within organizational contexts that increasingly emphasize systematized routines alongside individualized care [5]. This calls for a theoretical approach attentive to how meaning and action emerge through lived experience. Drawing on Merleau–Ponty’s phenomenology of perception, this study conceptualizes practice as grounded in embodied perception, through which meaning emerges within clinical interaction [6, 7].

The aim of this study was to explore and describe nurse managers’ practices during ward rounds in Japanese hospital settings, with particular attention to how perception guides moment‐to‐moment action in clinical encounters. Using a phenomenological approach informed by Merleau–Ponty’s theory of perception, this study examines how nurse managers enact clinical presence, respond to unfolding situations, and sustain relationships during everyday ward rounds.

## 2. Methods

### 2.1. Design

This study employed a phenomenologically informed qualitative design grounded in Merleau–Ponty’s phenomenology of perception to explore how nurse managers’ perception‐guided practices unfolded during ward rounds. This approach was appropriate for illuminating tacit dimensions of managerial practice as they emerged through lived experience and relational engagement in everyday clinical work [6, 7]. From this perspective, nurse managers’ practices during rounds were understood as emerging through situated perception and engagement with unfolding clinical situations, rather than as preplanned behaviors or purely cognitive decision‐making processes.

### 2.2. Participants and Setting

Participants were nurse managers working in wards of a university hospital in the Tokyo metropolitan area. The hospital was selected because nurse managers routinely conducted ward rounds as part of their everyday managerial practice, providing an appropriate setting for observing the phenomenon under study. Eligibility criteria included nurse managers with at least 2 years of experience in the role.

Participants were recruited through purposive sampling to identify nurse managers with relevant experience of ward‐round practices. After obtaining permission from the director of nursing at the study hospital, the first author explained the study verbally and in writing during a nurse manager meeting. Nurse managers who expressed interest in participation were subsequently provided with detailed information about the study and gave written informed consent before participant observation and interviews. Observation and interview schedules were then arranged individually with participants.

Five nurse managers participated in participant observation and interviews, and data from all five participants were analyzed. As the analysis progressed, the final in‐depth phenomenological analysis focused on three participants (A, B, and C), for whom the relationship between what was perceived during ward rounds and how those perceptions guided practice as situations unfolded could be analytically traced across the observational and interview data. In the remaining two cases, although both observational and interview data were analyzed, this perception–practice relationship could not be delineated with sufficient clarity to support interpretation of the specific phenomenon under investigation. The decision to focus the final analysis on three participants was therefore made during the analytic process and was not based on differences in the amount or richness of the data.

The three participants were nurse managers in their 40s–50s, each overseeing a surgical or medical ward.

### 2.3. Data Collection

Data were collected between February and June 2016 using repeated participant observations during ward rounds and phenomenological interviews guided by broad prompts. For each participant, four cycles of participant observation and phenomenological interviewing were conducted. For the three participants included in the final in‐depth analysis, this yielded 12 round situations in total. Participant observation allowed the first author to document visible, embodied practices—such as tone, pacing, facial expression, posture, positioning at the bedside, and micro‐interactions with patients—within the unfolding ward context.

Detailed fieldnotes were written following each observation. Fieldnotes included: (1) the date of observation, (2) descriptions of the overall ward atmosphere and contextual situation, and (3) detailed descriptions of nurse managers’ practices during rounds in chronological sequence. Observational descriptions focused particularly on situations that appeared meaningful or called for further phenomenological reflection during rounds. Fieldnotes were structured with analytic margins on both sides of the page. The left margin was used to record the researcher’s immediate impressions, emotional responses, and emerging interpretations related to observed situations. The right margin was used to document reflective questions directed toward the researcher’s own assumptions and interpretations. This process supported phenomenological reflection by preserving situational descriptions alongside the evolving interpretive dialog between observation and analysis.

Interviews were conducted to further explore perceptual meanings that were not fully accessible through observation alone, including what became salient for the nurse manager and how the situation was sensed and interpreted. Interviews were guided by broad phenomenological prompts rather than a fixed interview guide. Observation episodes informed subsequent interview questioning, allowing observational and interview data to be explored iteratively as participants reflected on specific moments occurring during rounds. Participants were invited to describe what they had perceived, attended to, or felt in particular situations, as well as how they understood their responses and interactions with patients.

Examples of interview prompts included the following: “What drew your attention to that patient first during the round?” “What stood out to you in that moment?” “What were you sensing when you removed your mask while speaking with the patient?” and “What was happening in the interaction when you shifted from using the patient’s family name to first name?” Additional prompts explored bodily responses, relational interactions, reflective experiences, and meanings attributed to particular clinical encounters.

In phenomenological research, interviewing is understood as a dialogical process through which experience becomes describable, rather than a simple retrieval of facts [7, 8]. Interviews lasted up to 60 min, were audio‐recorded with consent, and were transcribed verbatim for iterative interpretation alongside observational fieldnotes.

### 2.4. Data Analysis

Analysis followed an interpretive phenomenological process guided by Merleau–Ponty’s phenomenology of perception, particularly his account of the figure–ground structure of perception and the embodied nature of meaning [6]. Data from all five participants were included in the initial analytic process. Fieldnotes and interview transcripts were read repeatedly and examined together to identify round situations in which observed actions could be interpreted in relation to what nurse managers described as perceptually salient in those situations. As the analysis progressed, this relationship between perception and practice as situations unfolded could be analytically traced with sufficient clarity across the observational and interview data for three participants (A, B, and C), and the subsequent in‐depth phenomenological analysis focused on these three cases. The final analytic focus was therefore determined by the extent to which this perception–practice relationship could be traced across the combined data, rather than by differences in the amount or richness of data available for each participant. Sample adequacy was considered in relation to the study aim, the specificity of the phenomenon under investigation, and the explanatory value of the combined observational and interview data, consistent with the principle of information power [9]. Theoretical saturation was not used as a criterion for determining the final in‐depth analytic focus.

Each situation was then reconstructed as a thick description including patient context, the nurse manager’s actions, positioning, and the immediate ward atmosphere [10]. Interview data were examined to identify what the nurse manager described as perceptually salient (what became “figure”) and what receded into the background (“ground”), thereby illuminating how perception oriented actions within the situation. Observation episodes informed interview prompts, allowing participants to reflect on specific moments occurring during rounds and enabling a deeper understanding of how situations were perceived and responded to. Fieldnotes and interview transcripts were interpreted together to examine how embodied actions, spatial positioning, silence, tone, and relational interactions unfolded within particular situations. Through iterative comparison of observational and interview data, descriptions of perception and practice were integrated into coherent situational narratives (Figure 1). Finally, meanings across situations were clustered and interpreted into themes characterizing nurse managers’ perception‐guided practices during rounds. Interpretations were refined through repeated engagement with both forms of data to ensure that themes remained grounded in participants’ lived experiences and observed practices [7].

**FIGURE 1:**
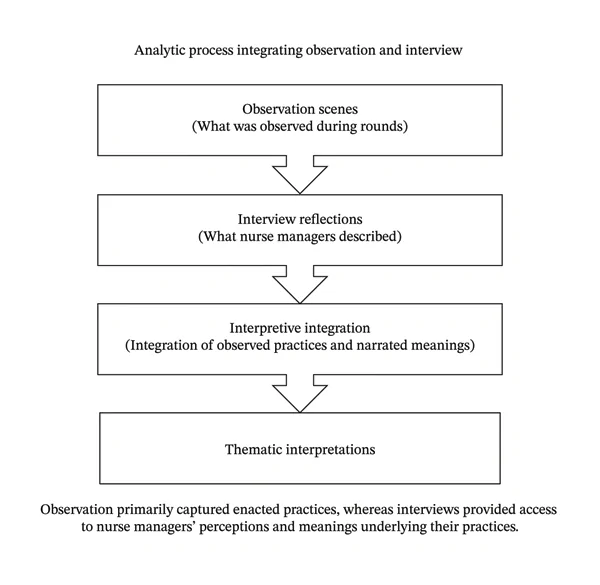
Analytic process for integrating observation and interview data.

### 2.5. Rigor

The first author had prior clinical experience as a nurse manager and had experience conducting ward rounds in clinical practice. This background supported sensitivity to the contextual, relational, and managerial dimensions of ward‐round practice, while also creating the possibility that prior assumptions could influence observation and interpretation. Reflexive attention to these assumptions was therefore maintained throughout data collection and analysis.

Rigor was strengthened through sustained engagement in the field, repeated participant observations, and iterative comparison between observational fieldnotes and interview transcripts. Credibility was supported by integrating observational and interview data to examine how practices unfolded within specific clinical situations. During the study period, the first author also sought feedback from participants regarding whether the descriptions resonated with their experiences. Dependability was enhanced through systematic documentation of fieldnotes and maintenance of an audit trail of analytic decisions throughout interpretation. Confirmability was strengthened by grounding interpretations in participants’ lived accounts and observed practices, while peer debriefing and reflexive discussions were used to critically examine the influence of the researcher’s prior assumptions and professional experiences. Transferability was supported through thick descriptions of ward contexts, interactions, and rounding situations, allowing readers to evaluate the relevance of findings to other clinical settings [11].

### 2.6. Ethical Considerations

This study was approved by the institutional research ethics committee (approval no. 3562) and conducted in accordance with the principles of the Declaration of Helsinki. The approved protocol identified nurse managers as the research participants and included participant observation of their routine ward rounds. The protocol also specified that information concerning patients encountered by nurse managers during rounds could be collected, that observational information would be recorded in a manner that did not permit identification of individuals, and that study findings would be disseminated through academic presentations and publications. Written informed consent was obtained from nurse manager participants before observation and interviews. Nurse managers were informed that they could decline to answer any questions or withdraw from the study at any time without penalty.

The original protocol did not explicitly specify a formal opt‐out procedure for patients or an explicit waiver of individual patient consent. In practice, before observation, nurse managers introduced the first author to patients as a doctoral student accompanying ward rounds for research purposes. Patients were informed of the researcher’s presence and were given the opportunity to decline observation; no patients declined observation during the study period. Patients were not interviewed or treated as direct research participants. To protect confidentiality, identifying information was removed from fieldnotes and transcripts, pseudonyms were used in reporting, and details of clinical situations were modified where necessary to prevent identification. Electronic research data were stored on a password‐protected computer that was not connected to external networks, and hard‐copy materials were kept in locked storage.

This study is reported in accordance with the Standards for Reporting Qualitative Research (SRQR) [12]; the completed SRQR checklist is provided as supporting information (available here).

## 3. Results

The final in‐depth phenomenological analysis focused on three nurse managers (A, B, and C), for whom the relationship between perception and practice could be analytically traced across the observational and interview data. They had between two and 6 years of experience in managerial roles and worked in acute care wards with differing clinical characteristics. Across these three participants, twelve round situations were included in the final analysis, capturing diverse moments of nurse managers’ interactions with patients during everyday practice. Participant characteristics are summarized in Table 1.

**TABLE 1:** Participant characteristics.

Participant	Age group/gender	Ward type (beds)	Nurse manager experience (years)	Total clinical experience (years)	Notes (context)
A	40s/Female	Cardiovascular surgery and cardiology ward with CCU (43 beds)	6	25	Also served as a nursing education coordinator; experience across orthopedic, ICU, private ward, and outpatient settings.
B	50s/Female	Mixed surgical ward with SCU (41 beds)	5	28	Also served as a nursing education coordinator; extensive ICU experience and rotations across multiple specialties.
C	40s/Female	Gastroenterology ward (52 beds)	2	28	High admission/discharge turnover; approximately half of staff had ≤ 4 years of experience.

Abbreviations: CCU, coronary care unit; ICU, intensive care unit; SCU, stroke care unit.

Analysis focused on how nurse managers’ practices during rounds were guided by what they attended to and perceived, and by the actions that emerged in response. By examining each scene in terms of what the nurse manager oriented toward, what was perceived as salient in the situation, and how practice unfolded, shared patterns of meaning were identified across cases.

Through this analytic process, four themes were generated that describe the perception‐guided practices identified in these cases. These themes illuminate distinct yet interrelated dimensions of nurse managers’ engagement with patients and the clinical environment, and together show how perception‐guided practice in the rounds examined. An overview of the themes and illustrative practices is presented in Table 2.

**TABLE 2:** Overview of themes and illustrative practices.

Theme	Core meaning	Illustrative practice
Creating Clinical Spaces of Presence	Relational clinical space emerging through attentive presence	Remaining with a patient in silence
Responding Through the Body	Perception expressed through embodied responsiveness	Reaching toward a patient or softening tone and expression
Reflecting in the Moment	Reflection and adjustment within unfolding encounters	Recognizing insufficient presence and adjusting practice
Demonstrating Managerial Presence	Managerial responsibility becoming perceptible through relational engagement	Inviting patients to bring concerns about staff and care directly to the nurse manager

Each theme is presented through observational scenes, interview reflections, and interpretive descriptions to illustrate how practice unfolded within specific clinical situations.

Interview excerpts are identified using participant, interview, and transcript line codes (e.g., C‐3‐7), indicating the participant, interview occasion, and starting transcript line number, respectively. Patients referred to in observational scenes are identified using pseudonyms (e.g., Mr. W, Ms. R) to protect confidentiality.

### 3.1. Theme 1: Creating Clinical Spaces of Presence

This theme foregrounds how, in the cases examined, a relational clinical space emerged and was sustained through the nurse managers’ ways of being with patients, allowing patients to remain as they were and, in time, begin to express their experiences. During the rounds examined, such clinical spaces emerged not through the nurse managers leading conversations or actively intervening, but through their orienting themselves toward the patient’s space and remaining attentively within it. Their practice was grounded in sensitivity to the patient’s way of being, the atmosphere of the room, and the evolving relational space between themselves and the patient.

#### 3.1.1. Observation Scene

Mr. W’s room was located relatively close to the nurses’ station and contained four beds: two positioned near the corridor on either side of the entrance and two positioned by the window further inside the room. Mr. W’s bed was located on the far right side near the window. When entering the room, the researcher could see that the curtain around Mr. W’s bed was drawn only between his bed and the adjacent patient’s bed. No curtain covered the foot of the bed. From the entrance, the researcher could see only the bed rail at the foot of the bed, but not Mr. W himself. The curtains around the other patients’ beds were fully closed, making it unclear whether anyone was present there.

The round began shortly after 11:00 a.m., and Nurse Manager C visited Mr. W first. Upon entering the room, Nurse Manager C walked directly toward Mr. W’s bed. It was only after approaching the foot of the bed that Mr. W became visible to the researcher. Mr. W was sitting upright on the bed facing the window, with what appeared to be a notebook resting on his knees as he wrote in it. Oxygen tubing extended from the central wall outlet toward him, and an intravenous infusion bag hanging from an IV stand was connected to him through infusion tubing.

While saying softly, “*I came to see how you were doing… I was wondering how your pain has been…,*” Nurse Manager C moved from behind Mr. W’s left side to a position in front of him and knelt between the bed and the window, slightly to his left. However, Mr. W did not respond. He continued looking down at the notebook on his lap and did not look at Nurse Manager C, who remained kneeling directly in front of him. Nurse Manager C again asked how the sores around his mouth were, but Mr. W still did not answer. Even after some time had passed, he remained silent, continuing to look down at the notebook without turning toward Nurse Manager C. A period of silence followed. Throughout this silence, Nurse Manager C remained kneeling beside him with a calm expression.

After some time, Mr. W quietly began to speak.

“I forgot the kindness people showed me… even their names and faces… I can’t do anything anymore… please help me… maybe I’m finished now… everyone worries about me… I’ve worked hard all my life, so I still have some strength… it’s pathetic… maybe I’ve reached my limit… maybe I can hold on another two years…”

Nurse Manager C listened without changing expression, occasionally nodding while maintaining eye contact with him. At that moment, the social worker responsible for Mr. W entered the room. Nurse Manager C stood up and moved toward the foot of the bed. Standing in front of Mr. W, the social worker explained that the date for his temporary home visit had been arranged, that a care taxi had been scheduled, that he would travel home in a wheelchair accompanied by a community visiting nurse, that rescue opioids would be brought along, and that he would return directly to the hospital afterward.

Mr. W responded, “*Once I unlock the door, I can manage things at home myself.*” Then, still facing the window, he said to the social worker, “*I said something terribly rude to you before.*”

After the social worker left the room, Nurse Manager C approached Mr. W again and said, “*I’ll make sure the primary nurse is informed about the outing.*” Mr. W quietly murmured, still facing the window, “*It’s just pathetic.*” Nurse Manager C then left the room.

#### 3.1.2. Interview Reflection

Nurse Manager C described Mr. W, whom she visited during rounds that day, as follows:

“*Mr. W had lung cancer, and he was someone who would not let anyone in. He had avoided hospitals for a long time, and by the time he was admitted, he was already physically at his limit. Even when visiting nurses went to his house, he would not let them enter. He would not answer phone calls from outpatient nurses either. He apparently had a brother somewhere, but he refused to tell anyone how to contact him and insisted that nobody should be contacted. The social worker, myself, and the palliative care team all tried to talk with him, but he kept saying the same thing: ‘Don’t contact anyone.’ Eventually we began asking ourselves whether there should also be cases like this — whether there are people who have lived their lives in this way and whose wishes should simply be respected. … He came to the hospital without even bringing money, and what he said was, ‘I just want to go home one more time.’ That was his final wish. So we started discussing who could accompany him, how to arrange a care taxi, and things like that. But we all felt that if we didn’t do it now, it might soon become impossible. Over the past few days his condition had improved significantly, the opioids were working, and he was able to sit up. We felt that if this was going to happen, it had to happen now—within this week.*” (C‐3‐7)

When Nurse Manager C entered the room, she described what immediately became salient to her:

“*I saw that he was awake… that he was able to remain sitting up… and I thought, ‘Ah, maybe he really can go out.’* ” (C‐3‐59)

While speaking to Mr. W, Nurse Manager C knelt beside him and remained there for some time. During this period, both Mr. W and Nurse Manager C remained silent. Reflecting on this silence, Nurse Manager C explained:

“*That was just his particular way of being. It was almost like he was saying, ‘Oh, so you came.’ I never felt that I was being rejected. I just hoped I could somehow enter his space naturally. So I stayed there thinking, ‘Maybe it’s okay for me to be here.’* ” (C‐3‐61)

Nurse Manager C further reflected on how her sense that it was “okay for me to be here” had gradually emerged through her ongoing interactions with Mr. W during his hospitalization:

“*At first things did not go well. Nurses kept trying different ways to get information from him. We were still trying to complete admission procedures, so I stepped in and suggested that perhaps we could obtain contact information through those procedures. But he became angry and said, ‘You’re the fifth person. The hospital only wants my money.’ That was how he interpreted it. I explained that wasn’t the case, and after that I continued checking in on him from time to time. He reacted similarly toward the social worker, saying things like, ‘You just care about money.’ But today, when the social worker came, he said, ‘I said something terribly rude to you before… but you’re still coming with me.’ That gave me the feeling that he had finally begun to feel reassured—that he now believed, ‘They are going to do what I asked for.’ I thought that perhaps the hospital had finally become a place where he could feel somewhat comfortable.*” (C‐3‐94)

#### 3.1.3. Interpretive Description

In this situation, Nurse Manager C entered Mr. W’s room seeking to personally understand whether he was physically able to go on the outing that represented his final wish. In doing so, Nurse Manager C perceived Mr. W sitting upright on the bed as “awake.” This perception did not appear merely as a recognition of his physical condition; rather, Nurse Manager C perceived Mr. W as being in a condition that made the outing possible.

At the same time, Mr. W did not immediately respond to Nurse Manager C’s greeting and continued silently looking down at his notebook. However, rather than interpreting this silence as rejection, Nurse Manager C sensed from Mr. W’s reactions and manner of being that it was “okay” for her to remain there.

Seeking to enter Mr. W’s space naturally, Nurse Manager C knelt beside him and remained there. Nurse Manager C did not attempt to immediately elicit conversation or intervene in the patient’s silence. Rather, she remained beside the patient while attending to subtle relational cues that signaled whether her presence was being accepted. Her practice unfolded through a situated sensitivity to the atmosphere of the room and the patient’s way of being. By remaining beside the patient without disrupting the silence, a relational space gradually emerged in which the patient began to speak.

Thus, in Creating Clinical Spaces of Presence, nurse managers did not rely on explicit verbal indications that their presence was being accepted by the patient. Rather, they perceived subtle cues within the patient’s way of being and continuously adjusted how they remained present within the patient’s space. Through this ongoing relational attunement, a shared clinical space emerged in which speaking could become possible.

### 3.2. Theme 2: Responding Through the Body

This theme foregrounds the embodied responsiveness through which, in the cases examined, what became perceptually salient to the nurse managers was expressed through their bodily engagement within clinical encounters. During the rounds examined, perception and bodily response were closely intertwined, sometimes before the meaning of what was perceived was consciously articulated. Gestures, posture, tone of voice, pacing, and movements toward the patient arose as part of the nurse managers’ bodily responsiveness to what they perceived in the moment. Through these bodily responses, reassurance and relational responsiveness became visible within the encounter.

#### 3.2.1. Observation Scene

On this day, Nurse Manager B began rounds at exactly 10:00 a.m. After visiting 13 patients in rooms near the nurses’ station, she entered Ms. R’s room. The room was a five‐bed room, with two beds on the right side and three on the left side from the entrance. Curtains surrounded all of the beds, and the room was so quiet that almost no sounds could be heard. Nurse Manager B first approached the bed in the back right corner by the window and opened the curtain while calling the patient’s name. At that moment, Ms. R responded, “*He’s in dialysis.*”

Immediately afterward, Nurse Manager B approached Ms. R’s bed on the left side by the window and opened the curtain, saying, “*I’m Nurse Manager B.*” Ms. R was sitting upright on the bed facing the window, writing something in a notebook placed on the overbed table. A single travel bag was placed on the floor beside the bed. Nothing else was on the overbed table, and the area around the bed was neatly organized.

Standing diagonally in front of Ms. R, Nurse Manager B said, “*It’s already been 2 weeks, hasn’t it?*” Ms. R then spoke about planning to send her belongings home by cash‐on‐delivery courier service and about how difficult it might be to travel to the Shinkansen station because rain was expected the next day. Suddenly, she turned toward Nurse Manager B and said, “*I had even thought about writing a will.*”

At that moment, Nurse Manager B took one or two steps toward Ms. R and leaned slightly forward as if peering into the face of the seated patient. Simultaneously, her left hand extended toward Ms. R’s back. Although the palm of her left hand remained open, it stopped just before touching her back. After this, Ms. R said nothing further. Nurse Manager B also remained silent, continuing to look at Ms. R while keeping her left hand extended toward her back.

After some time, Ms. R quietly said, “*Tomorrow I’m finally being discharged.*” After these words, Nurse Manager B slowly withdrew her hand from behind Ms. R’s back and said, “It’s been very hard for you, hasn’t it?” She then left the room.

#### 3.2.2. Interview Reflection

Nurse Manager B explained why she spoke to Ms. R by saying, “*It’s already been 2 weeks, hasn’t it?*” as follows:

“*I often go to see patients once their discharge has been decided. I might say something like, ‘So it’s finally been decided.’ Usually, I go because I want to create an opportunity for conversation. Sometimes patients say things like, ‘That was what I had been worried about,’ or ‘That was what had really been troubling me.’ Ms. R had been admitted to the ICU, so originally she was only expected to stay for a day or two. But because of bed availability, we had to ask her to stay another two or three days. Right after she returned to this ward, she stayed in a room close to the nurses’ station, and at that time we were able to talk quite a bit. But later she moved to a room farther away from the nurses’ station, and as her condition gradually stabilized, I think I left things more to the staff. It felt like, ‘Wow, two weeks have already passed.’* ” (B‐1‐44)

While speaking about her discharge scheduled for the following day, Ms. R suddenly said, “*I had even thought about writing a will.*” Reflecting on that moment, Nurse Manager B stated:

“*She didn’t really seem like the kind of person who would say something like that… I had gone to talk with her for the first time in a while, and then she suddenly said that. And I remember thinking, ‘Ah… yes, of course.’ I already knew that people who come here often carry many things with them. She was far from home, you know. She had come alone from a distant prefecture and was separated from her family.*” (B‐1‐27)

Nurse manager B further reflected:

“*Patients probably talk frequently with the staff nurses, but I think there are times when, within this particular distance between a nurse manager and a patient, patients end up saying something when the nurse manager comes by. When I think about it that way, hospitalization is just one scene within a person’s life, and perhaps there are things they had wanted to say during that particular moment.*” (B‐1‐60).

#### 3.2.3. Interpretive Description

Nurse Manager B explained that she often visited patients once their discharge had been decided, and on this day, she entered Ms. R’s room because she was scheduled to be discharged the following day. She perceived Ms. R sitting beside a single travel bag, with the area around her bed already neatly organized. This perception gave rise to her comment, “It’s already been 2 weeks, hasn’t it?” In that moment, Nurse Manager B appeared to perceive not only Ms. R as someone who had recovered sufficiently to be discharged, but also as someone who had lived through those 2 weeks of hospitalization.

In response, Ms. R suddenly stated, “I had even thought about writing a will.” Nurse Manager B immediately took one or two steps closer and extended her left hand toward Ms. R’s back.

What became salient to Nurse Manager B in this moment was not simply Ms. R as someone who would be discharged the following day, but Ms. R as someone who had lived through an experience serious enough to contemplate writing a will. Her left hand appeared to reach toward Ms. R as someone who had lived through that experience.

Although Ms. R’s statement surprised Nurse Manager B, it was not entirely incomprehensible to her. During the interview, however, Nurse Manager B did not explicitly refer to the movement of her left hand. Her lack of explicit reference to this movement suggests that the bodily response may not have been fully articulated in conscious reflection. The gesture appeared to emerge before its meaning was fully articulated in words. In this sense, her body responded to the patient’s lived experience before the meaning of the situation was fully expressed in words.

A different form of embodied responsiveness emerged when a nurse manager’s perception of a patient’s condition gave rise to reassurance. In such situations, this reassurance was expressed not through an overt reaching gesture, but through subtle bodily changes, such as tone of voice, facial expression, pacing, and manner of presence.

#### 3.2.4. Observation Scene

On this day, the round began with Mr. X. His room was a five‐bed room located across the hallway from the nurses’ station, the second room from the end. Mr. X’s bed was positioned near the entrance on the right side of the room, and he was sitting in a wheelchair facing the doorway. A nasogastric tube had been inserted, and part of his right forearm, visible beneath the sleeve of his long‐sleeved pajamas, was immobilized with a splint extending to the palm, with bandages wrapped to the fingertips. His left hand was also covered with bandages and protective netting, leaving his fingertips invisible.

As soon as Nurse Manager B entered the room, she immediately approached Mr. X and sat beside the wheelchair at eye level with him. She said, “*I came to see how you were doing after the surgery.*” Although Nurse Manager B usually spoke rather quickly, on this occasion her speech was slower and gentler than usual. Her facial expression also appeared softened.

Mr. X responded, “*I remember you. You came to see me before, didn’t you?*” He then suddenly began describing the circumstances that had led to his injury, saying, “*I did something foolish.*” While sitting beside the wheelchair, Nurse Manager B listened to Mr. X’s disclosure, occasionally nodding and offering brief verbal responses. After Mr. X finished speaking, Nurse Manager B told him that she had been worried because the surgery had been such a major operation. Mr. X then commented that the nurses on the ward had been very kind to him. Nurse Manager B replied, “*Thank you,*” and left the room.

#### 3.2.5. Interview Reflection

Mr. X had undergone reconstructive surgery for burns to both forearms on the previous day. Nurse Manager B explained why she visited Mr. X first during rounds that morning as follows:

“*He had surgery yesterday, and even before the operation his wife had been carrying a lot of worries. There had also been some developments the previous week, like him developing a fever… and then the surgery yesterday. I was also concerned about his wife. He was elderly, and the skin flap reconstruction had been extensive, so I was wondering how he was doing. That’s why I went straight to see him.*” (B‐3‐2)

In fact, before beginning rounds, Nurse Manager B had already noticed Mr. X sitting in the wheelchair.

“*When I was in the hallway that morning, I could already see that he was sitting up, and I thought, ‘Ah, his postoperative course must have gone well yesterday.’ I felt reassured that he was doing okay, so I went to see him feeling somewhat relieved.*” (B‐3‐7)

During the interview, Nurse Manager B reflected on why Mr. X had disclosed such personal feelings, remarking, “*It’s strange, isn’t it?*” (B‐3‐38) and “*I wonder what it was.*” (B‐3‐41). She then reflected on having seen Mr. X before rounds:

“*Usually when I go to see someone after surgery, I approach them feeling quite worried, wondering if they are really okay. But maybe this time I myself also felt somewhat relieved.*” (B‐3‐46)

During the interaction, Mr. X suddenly began speaking self‐reproachfully, saying, “*I did something foolish.*” Reflecting on that moment, Nurse Manager B stated:

“*I think maybe the surgery itself marked some kind of turning point for him. Usually when I go on rounds, conversations do not develop in that direction. I thought he might talk about postoperative pain or bleeding or something like that, but instead he began talking that way, and honestly that surprised me. Surgery, especially major surgery, is such a major event in a person’s life. At those moments, all kinds of memories probably come back into the patient’s mind. I felt like I saw another side of him.*” (B‐3‐19)

#### 3.2.6. Interpretive Description

Before entering the room, Nurse Manager B had already perceived Mr. X sitting upright in a wheelchair and sensed that he was “*doing okay.*” During the round, this perception was further reinforced through her encounter with him. Her tone of voice became gentler and slower than usual, and her facial expression softened. These changes suggest that her perception of reassurance was expressed bodily in the way she approached and engaged with Mr. X.

Mr. X subsequently began speaking about the circumstances surrounding his injury. Nurse Manager B’s response was not limited to assessing his postoperative condition; rather, her bodily manner of engagement reflected how she perceived him in that moment. In this situation, her sense of reassurance became visible bodily through subtle changes in tone, facial expression, pacing, and manner of presence.

### 3.3. Theme 3: Reflecting in the Moment

This theme foregrounds how, in the cases examined, the nurse managers reflected on and adjusted their own practice within unfolding clinical encounters. During the rounds examined, the nurse managers did not respond to patients’ words or situations in a predetermined manner. As interactions unfolded, they reconsidered not only how to respond to patients, but also their own involvement in the situation. Within these encounters, reflection did not occur separately from practice. Rather, it emerged within the encounter itself, as the nurse managers reconsidered and adjusted how they listened, questioned, and responded. The following situation involving Nurse Manager C illustrates how such reflection unfolded within an interaction marked by uncertainty and emotional tension.

#### 3.3.1. Observation Scene

On this day, Nurse Manager C visited Ms. Y first during rounds. Ms. Y’s room was a two‐bed room located across the hallway from the nurses’ station, the fourth room down the corridor and the second farthest from the station. Ms. Y’s bed was positioned at the back of the room near the window. She was lying on her right side facing the entrance. A central venous nutrition catheter had been inserted, and intravenous infusion was being regulated by an infusion pump.

When Ms. Y noticed Nurse Manager C entering the room, she said, “*Thank you.*”

Nurse Manager C approached Ms. Y, leaned slightly forward, and said, “*Were you able to sleep? I heard your fever went up and that you were feeling unwell.*” In response, Ms. Y began speaking in a strong tone about her frustration toward the attending physician and the medical team, saying, “*I don’t like that medical team. It feels like Dr. △△ is making decisions without really understanding me.*” Afterward, she spoke more slowly and carefully, choosing her words as she said, “*I want to speak properly with the senior physician.*”

Nurse Manager C listened while nodding repeatedly and saying softly, “*Mm, mm, mm.*” Ms. Y then quietly murmured, almost to herself, “*I wonder whether this treatment is really the right one.*” A brief silence followed, but Nurse Manager C remained beside her. Ms. Y again spoke in a strong tone, saying, “*They just keep doing things so quickly…*”

At that moment, Nurse Manager C asked, “*How is your leg?*” As she spoke, she moved toward the foot of the bed, lifted the blanket, and looked at Ms. Y’s leg. She then returned to Ms. Y’s bedside and said, “*I haven’t been able to come by often enough to listen to you.*” Ms. Y responded, “*No, you always encourage me.*” She further emphasized, “*The nurses here have all really taken such good care of me.*”

Nurse Manager C then asked about her family visits, saying, “*Is your father here?*” Ms. Y replied, “*Yes.*” Nurse Manager C responded, “*I’ll come again,*” and before leaving the room, she quietly organized Ms. Y’s personal belongings that had been scattered across the overbed table.

#### 3.3.2. Interview Reflection

Ms. Y had been hospitalized with suspected infectious enteritis. Although her room was located far from the nurses’ station, Nurse Manager C explained why she chose to visit Ms. Y first during rounds that day:

“*She had actually been in the hospital for quite a long time. It was infectious enteritis, but the diagnosis was still not entirely clear. She would develop fevers and have diarrhea… and things were not really improving. She was also dissatisfied with her attending physician, and at one point she became very upset with the staff about it. After I spoke with her, and after the senior physician also talked with her, she calmed down for a while. But then her condition worsened again, and it felt like those frustrations were beginning to resurface. Last night as well, she became upset about the treatment and responses from the younger doctors. The night nurse told me, ‘Last time she felt better after speaking with you, so she said she wanted to talk with you again today.’* ” (C‐4‐3)

Nurse Manager C had already been informed by the night nurse that Ms. Y wanted to speak about the physicians. As soon as Ms. Y noticed Nurse Manager C entering the room, she immediately said, “*Thank you.*” Reflecting on that moment, Nurse Manager C described Ms. Y as follows:

“*She had this expression like, ‘Ah, you finally came. I’ve been waiting for you.’ That was the feeling.*” (C‐4‐126)

While remaining beside Ms. Y, Nurse Manager C adopted a posture of listening attentively. At first, Ms. Y spoke strongly about her anger and dissatisfaction toward her attending physician. Gradually, however, her tone softened, and she began speaking more slowly, at times almost murmuring to herself. Reflecting on how she positioned herself while listening, Nurse Manager C stated:

“*When I’m talking with someone, I want them to feel that I’m really here listening to them properly. I need to be able to see their face, and they need to be able to see mine. So things like matching eye level, the expression on my face while listening, and the timing of my responses are very important to me…*” (C‐4‐151)

During the interaction, Nurse Manager C told Ms. Y that she had not been able to visit frequently enough or spend sufficient time listening to her. Reflecting on why she said this, Nurse Manager C explained:

“*Patients really are waiting for you… And in that moment I thought, ‘Ah… I messed up.’ I realized it was me. I felt that I was the reason.*” (C‐4‐129)

#### 3.3.3. Interpretive Description

Before entering the room, Nurse Manager C had already been informed that Ms. Y wished to speak with her. Thus, she approached the room expecting that Ms. Y might be waiting for her. When Nurse Manager C entered the room, Ms. Y immediately said, “*Thank you,*” and Nurse Manager C sensed that she had indeed been waiting for her.

At first, Ms. Y spoke strongly about her dissatisfaction and anger toward her physicians. However, as the interaction unfolded, her way of speaking shifted. At times, she spoke more slowly, murmuring quietly about whether the treatment was truly appropriate. Through these shifts in tone and expression, Nurse Manager C perceived that Ms. Y’s experience involved not only anger toward her physicians, but also uncertainty and distress.

While listening to Ms. Y, Nurse Manager C became aware that she had not visited often enough to hear her concerns. This awareness emerged within the interaction itself and led her to say, “*I haven’t been able to come by often enough to listen to you.*” Reflecting on this moment during the interview, Nurse Manager C stated, “*Ah… I messed up. I realized it was me. I felt that I was the reason.*”

In this situation, reflection emerged within practice rather than after the encounter. As Ms. Y’s expressions shifted between anger, uncertainty, and distress, Nurse Manager C reconsidered her own involvement and recognized that her own absence had become part of the situation. This recognition immediately altered her response: Rather than remaining solely in the role of listening to complaints about the physicians, she acknowledged to Ms. Y that she had not been sufficiently present to listen to her concerns. Reflecting in the Moment therefore describes how nurse managers questioned and adjusted their own practice while still engaged in the unfolding clinical encounter.

### 3.4. Theme 4: Demonstrating Managerial Presence

This theme foregrounds how, in the cases examined, the nurse managers’ managerial responsibility became perceptible through their clinical and relational engagement with patients. During the rounds examined, the nurse managers sometimes became explicitly recognized by patients as “the nurse manager.” In these situations, they positioned themselves as those who held responsibility for the ward by listening directly to patients and responding to concerns about staff and care. Through these interactions, managerial responsibility became perceptible while the nurse managers sustained reassuring relationships with patients.

The following situation involving Nurse Manager A illustrates how managerial presence became visible within an interaction with a patient who had repeatedly experienced distress and conflict with staff members.

#### 3.4.1. Observation Scene

As usual, Nurse Manager A began rounds with patients in the high care unit adjacent to the nurses’ station. She then proceeded to Mr. Z’s room, which was the second farthest room from the nurses’ station. The room was a two‐bed room, and Mr. Z’s bed was located near the window at the back of the room. At that time, the patient in the other bed closer to the entrance was absent.

When Nurse Manager A stood at the doorway, Mr. Z looked toward her. Nurse Manager A approached the bedside, knelt down to meet Mr. Z at eye level, and said, “*Good morning, Mr. Z. Yesterday was difficult too, wasn’t it? It came out again, didn’t it?*” while pointing toward Mr. Z’s nose. Mr. Z did not respond verbally. He had undergone a tracheostomy and had a cannula inserted. On the wall‐mounted panel above the bed were an oxygen flowmeter and suction bottle. Mr. Z’s personal belongings were scattered across the overbed table, and many personal items were also placed on the bedside cabinet.

Nurse Manager A pointed to an illustration placed on the bedside cabinet and asked, “*Who drew this? Your grandchild?*” The illustration depicted three people. Mr. Z’s expression brightened, and he replied, “*Yes.*” When Nurse Manager A said, “*They look just like you,*” Mr. Z smiled warmly and laughed softly, saying, “*Hehehe.*” Nurse Manager A then asked, “*Do your grandchildren visit you sometimes?*” and Mr. Z’s smile broadened even further.

At that point, Mr. Z looked at Nurse Manager A and asked, “*Are you the nurse manager?*” Nurse Manager A replied, “*Yes, I am,*” and then asked, “*How are the staff treating you?*” Mr. Z answered, “*They’re good.*” Nurse Manager A then lowered her mask slightly to show her face and said, “*If the staff ever do anything that troubles you, please let me know.*” Mr. Z smiled and replied, “*Thank you.*”

At that moment, Nurse Manager A’s hospital phone rang, and she left the room.

#### 3.4.2. Interview Reflection

On this day, before beginning rounds with Mr. Z, Nurse Manager A received a report from the night‐shift nurses regarding an incident involving him.

“*During the night-shift handover, the nurses told me that he had pulled out his gastric tube himself. They said they tried to restrain his hands, but he forcibly removed it anyway. This was not the first time—it was the second time. So I was talking with the night-shift leader about what might have been needed to prevent this from happening again.*” (A‐1‐90)

Against this background, Nurse Manager A visited Mr. Z during rounds. In the interview, Nurse Manager A described the background to Mr. Z’s behavior as follows:

“*He’s been hospitalized for a very long time following cardiovascular surgery, so it’s definitely a very stressful situation for him. Lately, he had often been scolding the staff, and when he couldn’t sleep, he would stay awake all night calling nurses over and criticizing them. I think there’s probably something behind it all. There were also times when he seemed delirious, so he had been taking psychiatric medication as well. And because he has a tube here*” *(pointing to her nose),* “*the discomfort is very strong. I think he also understands that if he pulls it out, the nurses will have trouble dealing with it. There’s a part of that too.*” (A‐1‐95)

Nurse Manager A further reflected on Mr. Z:

“*Mr. Z is someone who really watches people carefully. Earlier he said to me, ‘You’re the nurse manager, right?’ and I said, ‘Yes.’ Then when I asked, ‘Are the staff treating you well?’ he replied calmly, ‘Yes, they are.’ Of course, I know things are not actually that simple. But I think in that moment he was trying to smooth things over because his mood had improved. It’s interesting, isn’t it? The way he behaves with the staff can be quite different. Even while thinking, ‘I know the kinds of things you’ve been doing,’ he still responds to me in this way. But that’s also part of my role. I think it’s important that I intentionally go and listen to him. Sometimes I feel like I’m the one getting the best part of the interaction.*” (A‐1‐100)

#### 3.4.3. Interpretive Description

Before entering the room, Nurse Manager A was already aware of Mr. Z’s repeated incidents and his difficult interactions with staff members. Thus, she approached the encounter with an awareness not only of Mr. Z’s situation, but also of the difficulties arising in the relationship between him and the staff caring for him. However, when Nurse Manager A entered the room, Mr. Z did not appear merely as a patient associated with incidents or problematic behaviors. Through their interaction, Nurse Manager A perceived Mr. Z as someone who responded to her differently than he had reportedly responded to staff members.

When Mr. Z asked, “*Are you the nurse manager?*” Nurse Manager A perceived that Mr. Z recognized her not simply as another nurse, but as someone holding responsibility for the ward. Following this interaction, Nurse Manager A lowered her mask and showed her face while telling Mr. Z, “*If the staff ever do anything that troubles you, please let me know.*” Although staff members were in fact experiencing difficulties in responding to Mr. Z, Nurse Manager A positioned herself as the person to whom concerns about staff and care could be brought directly.

She did not initiate the interaction by asserting her managerial role; rather, her managerial presence became perceptible within the exchange itself. By responding as the person responsible for the ward and inviting Mr. Z to bring concerns about staff and care directly to her, Nurse Manager A enacted managerial responsibility through the relationship while maintaining a reassuring connection with him.

## 4. Discussion

### 4.1. Overview

This study explored nurse managers’ perception‐guided practices during ward rounds in Japanese hospital settings through Merleau–Ponty’s phenomenology of perception. The findings suggest that, in the cases examined here, nurse managers’ rounds may be understood not simply as routine managerial activities, but as relational and embodied practices through which the nurse managers remained responsive to patients and the clinical environment in situated ways. Rather than being enacted through standardized managerial behaviors, these practices unfolded through dynamic interactions among nurse managers, patients, and the ward environment, where meanings emerged within moment‐to‐moment encounters [6, 7].

In the cases examined here, rounds appeared to involve not only perceiving and responding to what was already visible, but also remaining open to what might emerge through the encounter itself. In this sense, rounds involved a situated attentiveness through which meanings and relationships emerged and managerial responsibility became perceptible within clinical encounters.

Existing research has examined nurse manager rounding from complementary perspectives. A systematic review of Nurse Manager Intentional Rounding found that previous studies had primarily examined the structure and frequency of rounding and outcomes, such as patient satisfaction and aspects of care quality, while also noting limitations in methodological quality and theoretical grounding [13]. In Japan, Shibuya et al. [14] used semistructured interviews with 10 head nurses to identify the purposes of patient rounds and their application to nursing management, including the use of information arising from head nurses’ intuition and awareness to inform care and improve patient satisfaction. Building on these contributions, the present study focuses less on the intended purposes, structure, implementation, or measured outcomes of rounding and more on how perception and practice were related as particular clinical encounters unfolded. Through repeated participant observations combined with phenomenological interviews, the in‐depth analysis of three nurse managers enabled examination of embodied, relational, and moment‐to‐moment dimensions of practice that are less readily captured through outcome‐focused designs or retrospective interview accounts alone.

Importantly, the four themes presented in this study do not represent a linear sequence of practice. Rather, they illuminate distinct yet interrelated dimensions of nurse managers’ engagement, including creating relational spaces, responding through the body, reflecting within unfolding encounters, and demonstrating managerial presence. Together, these findings suggest that nurse managers may sustain clinical presence not through oversight alone, but through ongoing relational responsiveness within patient encounters. Such a relational orientation is consistent with nursing leadership literature linking relational leadership approaches with positive nursing workforce and work‐environment outcomes [15] and nursing leadership with patient outcomes [1].

### 4.2. Theme 1: Creating Clinical Spaces of Presence

The findings suggest that, in the cases examined, creating clinical spaces of presence involved more than establishing opportunities for communication. Rather, the nurse managers’ rounds supported the emergence of relational spaces through interactions with patients, within which patients could begin speaking in ways that reflected their own pace and readiness. Such spaces did not emerge through directing conversation or leading care, but through ways of remaining present that allowed relational meanings to unfold gradually within the encounter. In this sense, presence in these encounters functioned as a form of “being‐with” that enabled patients’ expressions to emerge through silence, timing, and relational engagement rather than through questioning alone. This understanding resonates with phenomenological perspectives that conceptualize clinical encounters as relational events in which meaning emerges between self and other [6, 16].

In nursing scholarship, *presence* has long been recognized as a core dimension of caring and therapeutic engagement, often described as attentiveness, receptivity, and embodied availability [3, 17]. However, the present findings extend this understanding by suggesting that, within the rounds examined, presence functioned as a relational practice through which clinical spaces could emerge, allowing patients to express experiences that might otherwise remain unspoken within brief clinical encounters.

These findings also draw attention to the significance of nurse managers’ proximity to patients alongside their administrative responsibilities. While nursing leadership scholarship has often emphasized relationships between leadership and patient outcomes [1], the present findings suggest that clinical presence during rounds may provide one means through which patients’ lived experiences can emerge within everyday ward care.

Recent nursing leadership scholarship has emphasized leadership presence as an important dimension of nursing leadership with implications for patient, staff, and organizational outcomes [2]. The present findings extend this understanding by suggesting how, in the cases examined, leadership presence was enacted through relational engagement and being‐with patients within everyday ward‐round practice.

### 4.3. Theme 2: Responding Through the Body

The second theme suggests that, in the cases examined, perception and bodily responsiveness were closely intertwined within the interaction itself. What became perceptually salient to the nurse managers was manifest through facial expression, tone of voice, pacing, bodily positioning, and spontaneous movement toward the patient. At times, bodily responsiveness emerged before the meaning of what was perceived was fully articulated in words. This aligns with Merleau–Ponty’s view that perception is inherently embodied and action‐oriented: to perceive is already to be oriented toward the world and toward possible action, before reflective thought takes shape [6].

Embodied responsiveness did not appear in a single form. In some situations, it emerged suddenly, before its meaning was fully articulated in words, as illustrated by Nurse Manager B extending her hand toward Ms. R. In others, it appeared through subtle changes in tone of voice, facial expression, pacing, and manner of engagement. These findings suggest that the rounds examined involved more than observing patient conditions and subsequently deciding how to respond. Rather, perception, understanding, and response were intertwined within situated bodily engagement. This interpretation resonates with Benner’s account of clinical knowing as embedded in situated practice, in which skilled action may precede explicit analytical reasoning [18].

From a practice perspective, these findings suggest that, in the cases examined, the nurse managers’ responsiveness during rounds was expressed not only verbally but also through bodily ways of engaging with patients. Such embodied responsiveness may be an important, though often tacit, dimension of nurse managers’ direct clinical involvement. Recent nursing management scholarship has emphasized relational leadership behaviors as important for staff work engagement [19]. While this literature has primarily focused on leader–staff relationships, the present findings broaden attention to the relational dimension of nurse managers’ practice by illustrating how, in the cases examined, responsiveness was also enacted bodily in direct encounters with patients during rounds.

### 4.4. Theme 3: Reflecting in the Moment

The third theme suggests that, in the cases examined, nurse managers’ rounds involved more than understanding patients’ situations. Encounters with patients also became occasions through which the nurse managers reconsidered their own involvement within the unfolding situation. They perceived not only patients’ dissatisfaction, uncertainty, or distress, but also how their own absence, delay, or insufficient responsiveness had become part of the situation. In phenomenological terms, this process may be illuminated by Merleau–Ponty’s notion of reversibility, in which perceiving is not simply a one‐directional act but involves the perceiver being affected by and implicated in the perceptual situation [20]. In these encounters, the nurse manager’s own practice thus became perceptible to herself.

This “in‐the‐moment” reflection resonates with Schön’s concept of reflection‐in‐action, in which practitioners think within the unfolding situation rather than only after it has ended [21]. The present findings suggest, however, that in the cases examined, reflection was not solely an internal cognitive activity. Rather, it emerged within direct engagement with patients as the nurse managers reconsidered and adjusted their practice while the encounter was still unfolding. This capacity to be affected by the situation and to modify one’s response within the unfolding encounter may represent one dimension of nurse managers’ clinical expertise during rounds [7, 18].

Importantly, the findings from these cases suggest that nurse managers’ expertise may involve more than making timely judgments or decisions. It may also involve remaining open to having one’s own practice questioned through encounters with patients and being willing to re‐orient that practice within the moment. In this sense, encounters with patients may function not merely as opportunities for assessment, but as occasions through which nurse managers come to understand their own practice differently, consistent with phenomenological perspectives emphasizing how self‐understanding can be shaped through relations with others [16]. Recent nursing scholarship has emphasized reflective practice as an important process for learning from clinical experience and improving professional practice [4]. The present findings extend this perspective by suggesting that professional development for nurse managers may benefit from attending not only to retrospective reflection, but also to the perceptual awareness that supports reflection within unfolding clinical situations during rounds.

### 4.5. Theme 4: Demonstrating Managerial Presence

Theme 4 suggests that, in the cases examined, nurse managers’ rounds involved more than making their managerial role visible within patient encounters. Rather, the rounds examined functioned as practices through which the nurse managers became recognizable as those who held responsibility for the ward. In the observed encounter, managerial presence became visible when the patient explicitly recognized Nurse Manager A as “the nurse manager,” and she responded by inviting him to bring concerns about staff and care directly to her. In this way, the nurse manager did not simply assert authority; she enacted managerial responsibility through her clinical and relational engagement with the patient. This finding resonates with nursing leadership scholarship that has emphasized leadership presence as an important dimension of nursing leadership [2].

From a phenomenological perspective, managerial presence became perceptible through situated interaction rather than through role declaration alone. Nurse Manager A’s proximity, eye‐level positioning, and willingness to receive the patient’s concerns helped make her responsibility for the ward perceptible within the encounter. These practices may be understood phenomenologically as expressions of embodied and situated engagement [6]. Within such engagement, managerial responsibility became perceptible within the encounter. The present finding suggests that managerial presence may draw on clinical and relational capacities, while these capacities may take on a distinctive meaning when enacted within the nurse manager’s responsibility for patients, staff, and care within the ward.

In this case, demonstrating managerial presence was not simply a declaration of role, but a practice through which the presence of someone responsible for the ward became perceptible within the clinical space. By inviting the patient to bring concerns about staff and care directly to her, Nurse Manager A enacted this responsibility while maintaining a reassuring relationship with the patient.

### 4.6. Integrative Interpretation and Implications for Nursing Management

Taken together, the four themes suggest that nurse managers’ rounds may be understood not simply as supervisory activities, but as relational managerial practices through which nurse managers may sustain the relational world of the ward. Rather than functioning as discrete skills or techniques, the practices identified in this study suggest a dynamic process in which perception, embodiment, reflection, and responsibility were intertwined in the bedside managerial practices examined in this study. The findings further suggest that clinical and relational capacities may remain integral when nurses assume managerial roles; when enacted within the nurse manager’s responsibility for patients, staff, care, and the ward as a whole, these capacities may take on a distinctive meaning. This perspective extends existing understandings of nurse managers’ roles by suggesting that managerial practice unfolds not only through decision‐making [22], but also through situated and perceptual engagement with patients and the clinical environment.

From a theoretical standpoint, the findings contribute to nursing scholarship on presence by suggesting that presence may be understood not simply as a personal trait or a static quality of interaction, but as a relational and embodied phenomenon that unfolds through practice. By drawing on phenomenological concepts, such as intentionality, embodiment, and reversibility, this study illustrates how, in the cases examined here, perception‐guided practices gave rise to moments in which clinical relationships were initiated, deepened, questioned, and sustained, while managerial responsibility became perceptible within situated encounters. In doing so, the study bridges phenomenological theory and nursing management scholarship by offering a phenomenologically grounded understanding of managerial presence as lived practice [2], rather than as a set of leadership attributes alone.

The findings of the present study also informed subsequent development of the Nurse Manager Practices in Rounds Scale (NMPR). In that later study, findings from this phenomenological analysis were used as one source for candidate‐item generation, together with a review of the Japanese literature and interviews with nurse managers. Ten candidate items were initially derived from the present study, four of which remained in the final 22‐item scale following item reduction and psychometric evaluation. The two studies did not share participant‐level data: the subsequent NMPR study used separate samples and independently collected quantitative data. Thus, the relationship between the studies reflects conceptual continuity and subsequent operationalization of aspects of nurse manager practice rather than reuse of the empirical data analyzed in the present study.

Although the data were collected in 2016, the context of nursing management has changed substantially over the past decade. The COVID‐19 pandemic intensified expectations for nurse managers to respond to uncertainty, support staff, adapt rapidly to changing conditions, and sustain care delivery during crisis [23]. Contemporary nurse managers also face staffing pressures, including nurse shortages, skill‐mix challenges, resource constraints, and increasing care needs, all of which complicate nurse staffing decisions [24]. At the same time, digital transformation has increasingly reshaped nursing management, with growing emphasis on informatics competence, e‐leadership, and technology‐enabled coordination [25, 26]. These developments may both constrain opportunities for direct clinical presence and alter the conditions under which such presence is enacted. Accordingly, the specific forms of practice observed in these cases in 2016 may not be directly transferable to contemporary nursing management contexts. Nevertheless, the relational and perceptual dimensions illuminated in this study may remain relevant within these changing conditions. Direct engagement with patients during rounds may continue to provide opportunities for nurse managers to support the emergence of relational spaces, respond through embodied engagement, reflect on practice as encounters unfold, and make managerial responsibility perceptible within the clinical environment.

The findings also have implications for nursing management and professional development. They suggest that professional development for nurse managers may benefit from extending beyond cognitive judgment and managerial techniques to include attention to perceptual sensitivity, embodied responsiveness, and the capacity for reflection within action. Educational and professional development programs may benefit from incorporating opportunities for nurse managers to examine how their bodily presence, timing of engagement, and relational positioning shape clinical encounters during rounds. Organizational environments that support opportunities for reflection, relational engagement, and sustained clinical presence may also facilitate the development of these managerial practices.

Furthermore, understanding managerial presence as the capacity to sustain clinical space, rather than primarily in terms of visibility or control, invites a relational understanding of managerial practice. The nurse managers in this study enacted presence through restraint as well as action, maintaining managerial responsibility without necessarily directing the encounter. These findings suggest that managerial presence may be understood as a relational responsibility enacted through engagement with patients and the everyday clinical life of the ward.

### 4.7. Limitations

This study has several limitations. First, although data from all five participating nurse managers were included in the initial analysis, the final in‐depth phenomenological analysis focused on three nurse managers. In the remaining two cases, although both observational and interview data were analyzed, the focal relationship between perception and practice could not be delineated with sufficient clarity to support the same level of in‐depth phenomenological interpretation. In addition, the study was conducted at a single university hospital in Japan. The findings therefore reflect a limited number of contextually situated cases and may have been shaped by organizational culture, ward specialty, staffing conditions, and the professional histories of individual nurse managers. Second, although repeated observations and interviews enabled close attention to perception‐guided practice as it unfolded, the presence of the researcher during rounds may have influenced participants’ interactions. Third, the analysis focused on specific rounding situations observed during a defined period and does not capture how nurse managers’ practices may shift over time or across different organizational circumstances. In addition, because the data were collected in 2016, subsequent changes in healthcare and nursing management contexts—including postpandemic demands, staffing pressures, and digital transformation—may limit the transferability of the specific forms of practice observed in this study to contemporary settings. Accordingly, the findings should be understood as contextually situated, with their transferability to other practice contexts requiring consideration of contextual similarities and differences rather than statistical generalization. Future research across diverse settings and using longitudinal designs may further examine how, and under what conditions, the relationship between perception and practice can be discerned within nurse managers’ rounds, and how such practices relate to patient, staff, and organizational experiences.

## 5. Conclusion

This phenomenologically informed qualitative study illuminated how, in the cases examined, practices during ward rounds were guided by embodied perception in a Japanese hospital setting. Across four themes—Creating Clinical Spaces of Presence, Responding Through the Body, Reflecting in the Moment, and Demonstrating Managerial Presence—the practices examined emerged not as a set of standardized behaviors, but as situated and relational practices arising within unfolding clinical encounters. In these cases, the rounds examined involved more than routine surveillance; they were enacted as a form of clinical presence through which the nurse managers supported the emergence of relational spaces with patients, responded through embodied engagement, reflected within ongoing encounters, and made managerial responsibility perceptible within clinical encounters.

By making visible the perceptual, embodied, reflective, and relational dimensions of ward rounds, this study suggests that direct engagement with patients may constitute a meaningful dimension of everyday managerial practice. The findings contribute to nursing management scholarship by suggesting that managerial presence may be understood not simply as visibility or a leadership attribute, but as a situated and relational practice through which nurse managers may sustain engagement with patients and enact responsibility within the clinical environment.

## Author Contributions

Yoshie Tejima conceptualized and designed the study, conducted data collection and analysis, and drafted the manuscript. Maki Taniyama contributed to the interpretation of the findings and critical revision of the manuscript.

## Funding

This study received no external funding and was self‐funded.

## Disclosure

All authors approved the final version of the manuscript.

## Conflicts of Interest

The authors declare no conflicts of interest.

## Supporting Information

Additional supporting information can be found online in the Supporting Information section.

## Supporting information


**Supporting Information** The SRQR reporting checklist.

## Data Availability

The data that support the findings of this study are not publicly available due to ethical restrictions and the need to protect participant confidentiality.
